# Dynamical evolution of anisotropic response of type-II Weyl semimetal TaIrTe_4_ under ultrafast photoexcitation

**DOI:** 10.1038/s41377-021-00546-1

**Published:** 2021-05-14

**Authors:** Xiao Zhuo, Jiawei Lai, Peng Yu, Ze Yu, Junchao Ma, Wei Lu, Miao Liu, Zheng Liu, Dong Sun

**Affiliations:** 1 International Center for Quantum Materials, School of Physics, Peking University, 100871 Beijing, China; 2 State Key Laboratory of Optoelectronic Materials and Technologies, School of Materials Science and Engineering, Sun Yat-sen University, 510275 Guangzhou, Guangdong China; 3 Centre for Programmed Materials, School of Materials Science and Engineering, Nanyang Technological University, Singapore, 639798 Singapore; 4 Institute of Physics, Chinese Academy of Sciences, 100190 Beijing, China; 5 State Key Laboratory of Precision Measurement Technology and Instruments, School of Precision Instruments and Opto-electronics Engineering, Tianjin University, No. 92 Weijin Road, 300072 Tianjin, China; 6 Songshan Lake Materials Laboratory, 523808 Dongguan, Guangdong China; 7 Collaborative Innovation Center of Quantum Matter, 100871 Beijing, China

**Keywords:** Infrared spectroscopy, Optical physics

## Abstract

Layered type-II Weyl semimetals, such as WTe_2_, MoTe_2_, and TaIrTe_4_ have been demonstrated as a supreme photodetection material with topologically enhanced responsivity and specific sensitivity to the orbital angular momentum of light. Toward future device applications with high performance and ultrafast response, it is necessary to understand the dynamical processes of hot carriers and transient electronic properties of these materials under photoexcitation. In this work, mid-infrared ultrafast spectroscopy is performed to study the dynamical evolution of the anisotropic response of TaIrTe_4_. The dynamical relaxation of photoexcited carriers exhibits three exponential decay components relating to optical/acoustic phonon cooling and subsequent heat transfer to the substrate. The ultrafast transient dynamics imply that TaIrTe_4_ is an ideal material candidate for ultrafast optoelectronic applications, especially in the long-wavelength region. The angle-resolved measurement of transient reflection reveals that the reflectivity becomes less anisotropic in the quasi-equilibrium state, indicating a reduction in the anisotropy of dynamical conductivity in presence of photoexcited hot carriers. The results are indispensable in material engineering for polarization-sensitive optoelectronics and high field electronics.

## Introduction

Topological semimetals have recently been considered as potential material platforms to realize high-performance devices in electronics and optoelectronics due to their high carrier mobility^1–3^, broadband optical response^3–6^, and exotic topological properties^7–11^. Among the few topological semimetals that have been discovered, TaIrTe_4_ is a confirmed layered type-II topological Weyl semimetal, to have the effective charge separation from a topologically enhanced shift current response from the Weyl cones^12^, hence is a good potential material for future high-performance photodetection of low energy photons^5,12,13^. It has also been demonstrated in a recent work that a photodetection device based on WTe_2_, another layered type-II Weyl semimetal sharing the same C_2v_ lattice structure with TaIrTe_4_^14^, is sensitive to both spin angular momentum and orbital angular momentum of light^15^, which enables novel detection capability. Besides topological features, these materials have special anisotropic conductivities in *ab* plane as a result of the zigzag metal-metal chains along the crystallographic a-direction in layered Weyl semimetals with C_2v_ lattice structure, including TaIrTe_4_ and Mo(W)Te_2_^14^. The conductivity anisotropy provides an additional degree of freedom in developing polarization-sensitive optoelectronics devices^14^. Such anisotropic response is very similar to its two-dimensional layered counterparts, black phosphorus (BP) and ReS_2_^16,17^, but the optical responses of semimetals are capable of covering longer optical wavelength regions without the limitation from an energy gap.

In the vision of the potential ultrafast device applications, the study on dynamics of photoexcited hot carriers, i.e., electrons with elevated temperature comparing to the lattice temperature because of the high-field acceleration^18,19^, is indispensable. This is because the excited carriers in quasi-equilibrium distributions play the leading role in the transport of ultrafast devices and significantly influence the device performance. An established experimental approach for such studies is to optically excite the sample with ultrafast pump pulse, then a probe pulse arriving at different delays is used to probe the dynamical evolution after the pump excitation. When a polarization-resolved probe pulse is used, the anisotropic response of the studied materials in presence of hot carriers can be resolved with ultrafast time resolution.

In this work, pump–probe reflection spectroscopy is performed to study the dynamical response of TaIrTe_4_ under photoexcitation. A remarkable finding is that the anisotropic response of TaIrTe_4_ degrades in the quasi-equilibrium states after photoexcitation, in stark contrast with BP, a representative anisotropic semiconductor^16,20^. The reflection spectroscopy with different pump polarization indicates that the degradation of anisotropy is independent of the pump polarization. In addition, we also discuss the relaxation dynamics of the photoexcited carriers that can be fitted by tri-exponential decays. The initial ultrafast transient relaxation of photoexcited carriers supports that TaIrTe_4_ is an ideal material candidate for ultrafast optoelectronic devices, such as saturable absorber^21^, optical modulator, and switch^22,23^, that are capable of operating in mid-/far-infrared and terahertz region.

## Results

**Fig. 1:**
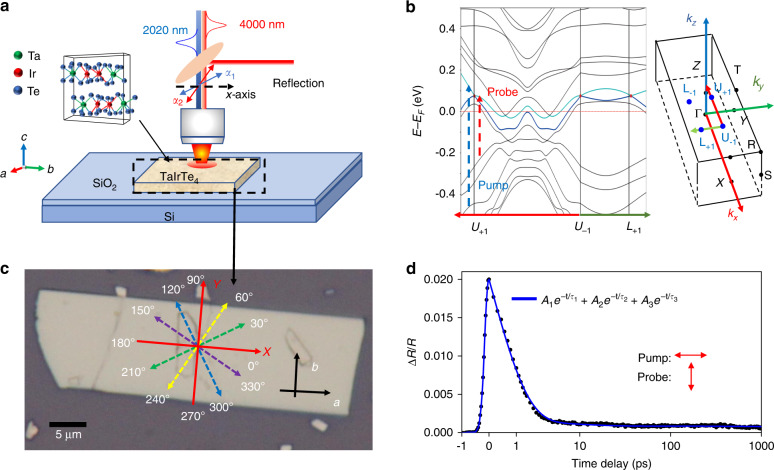
Experimental scheme, lattice and band structure, optical image, and typical transient reflection spectrum. **a** Experimental scheme of pump-probe spectroscopy. **b** The two energy bands (blue and purple) nest four type-II Wyle points, *U*_*+*_*, U*_*–*_, *L*_*+*_, and *L*_*–*_, near the *Γ* in the Brillouin zone. Band diagram sampled along the *U*_*+*_*-U*_*-*_-*L*_*+*_ direction, of TaIrTe_4_ with the typical pump and probe photon transitions marked by blue and red arrows, respectively. **c** Optical micrograph of the TaIrTe_4_ sample. **d** Transient reflection spectrums with cross-polarized pump (2020 nm) and probe (4000 nm) geometry. The curve is fitted by Δ*R/R* = $A_1e^{ - t/\tau _1} + A_2e^{ - t/\tau _2} + A_3e^{ - t/\tau _3}$ with *τ*_1_ = 1.02 ± 0.01 ps, *τ*_2_ = 4.04 ± 0.05 ps and *τ*_3_ = 14.0 ± 1.8 ns (blue solid line). The temperature is 297 K and the pump power is 300 μW

The crystal structure of the layered TaIrTe_4_ is orthorhombic (Fig. 1a), and the lattice constants related to the three crystallographic axes (*a*, *b*, *c*) are 3.770, 12.421,13.184 Å, respectively. TaIrTe_4_ can be derived from the WTe_2_ structure by doubling its *b-*axis and constructing the metal-metal chains by alternating Ta and Ir atoms, as shown in Fig. 1a. The zigzag chains are ordered in the [Ta-lr-lr-Ta] fashion when the chains are linked to form an extended planar layer, hence doubles the *b-*axis^14^. TaIrTe_4_ hosts only four type-II Weyl nodes, the minimal number imposed by symmetry in an inversion-break WSM^13^, thus it provides the simplest model of a WSM with broken inversion symmetry. Figure 1b shows the energy band structure of TaIrTe_4_. The calculation result shows that there are four energy bands around the Fermi level and the bands located at *Γ* nest together when taking the spin-orbit coupling effect into account. The electron and hole pockets touch and cross each other near the*Γ* and, therefore, form four type-II Weyl cones, the touching points of which are known as type-II time-reversal invariant Weyl points at *k*_*x*_ = 0.1190 *k*_*y*_ = 0.1865 in the Brillouin zone. It is notable that both the electron and hole pockets are fairly asymmetric along the *k*_*x*_ and *k*_*y*_ direction near the *Γ* point: the bands along *k*_*x*_ are much steeper than those along *k*_*y*_ as a result of the real space structural asymmetry, similar to the report in the existing literatures^14^. The Fermi level lies ~80 meV below the Weyl nodes as predicted by theory and verified by recent experiment^13^. Figure 1c shows an optical micrograph of the TaIrTe_4_ flake. The measurement is performed on a 100 nm thick flake at room temperature. The major layers of the relatively thick flakes are well protected by the surface, so the results reported in this work represent the features of bulk TaIrTe_4_ in the Weyl semimetallic phase.

Figure 1a shows the scheme of optical measurement. The TaIrTe_4_ sample is excited by a 614 meV linear polarized pump laser. Due to the photoexcitation, the optical conductivity of TaIrTe_4_ is modified. A 310 meV probe laser is used to detect the change of reflection (Δ*R*) after the excitation. The 310 meV probe photon energy is intentionally selected to match the wavelength at which topological enhanced photocurrent response was experimentally observed in TaIrTe_4_^12^, while the 614 meV pump photon energy is the idler of the optical parametric amplifier outputs that are used for differential frequency generation of the 310 meV probe photons (see “Materials and Methods” section). To study the dynamical anisotropic response of TaIrTe_4_, the probe polarization is rotated by a half-wave plate. The pump polarization is also adjustable, to study the anisotropic absorption of the pump light. The *x*-axis of polarization angle is set along the crystallographic *a*-axis (Fig. 1c) and the time resolution of the dynamical evolution is limited by the 300 fs laser pulse width.

Figure 1d shows representative transient reflection evolution with cross-polarized pump–probe geometry. The peak transient signal at time zero (Δ*R*/*R*|_*t*=0_) is positive, which implies a reflection increase after pump excitation. Then, Δ*R*/*R* decays exponentially, but it stays above zero even after a 1-ns delay. We have tried to use both bi-exponential and tri-exponential decay functions to fit the decay curves and the detailed fitting information can be found in Supplementary S1. It is clear that a tri-exponential decay function fits much better than a bi-exponential decay function, especially for the 2–5 ps region, suggesting there are three decay processes contributing to the relaxation of photoexcited carriers. While the slowest decay time constants are similar for both bi-exponential and tri-exponential models, the initial fast decay processes are fitted much better with two separate decay time constants than a single decay time constant, which indicates the multiple electron-phonon couplings are taking place with distinctive coupling strengths. After the pump excitation, the photoexcited carriers thermalized through a rapid carrier–carrier scattering process and reached a thermal equilibrium among carriers within the pulse width. The electron temperature after the thermal equilibrium is estimated to be about 2000 K taking 300 μW pump power focused on an 8 µm diameter pump spot [See Supplementary S7 for the deduction of the electron and lattice temperature]. After that, the hot carriers cool down through scattering with lattice. According to the above observations, we would propose the two fast decay constants *τ*_1_ and *τ*_2_, on the order of picosecond, are both related to electron-phonon scatterings. The faster component *τ*_1_ is attributed to the high-energy optical phonon scattering process and *τ*_2_ is attributed to the lower energy optical phonon and acoustic phonon scattering process. After the cooling through phonon scattering, the heat dissipation from TaIrTe_4_ to substrate contributes to the slowest relaxation process *τ*_3_, on the order of a nanosecond. The energies of major high-energy optical phonon are measured to be 12 meV to 27 meV according to Raman measurements reported in the literature^24^. The difference between *τ*_1_ and *τ*_2_ is much smaller in TaIrTe_4_ than that in semimetallic graphene and graphite^19,25^ because the optical phonon energy is far larger in graphene and graphite^26^. However, the better fitting results with tri-exponential comparing to bi-exponential verifies the high energy optical-phonon scattering is still distinguishably faster than low energy optical-phonon and acoustic-phonon scattering in TaIrTe_4_. The two dominant relaxation processes characterized by *τ*_1_ and *τ*_2_ are on the order of picosecond, which is good for ultrafast optoelectronic applications.

**Fig. 2:**
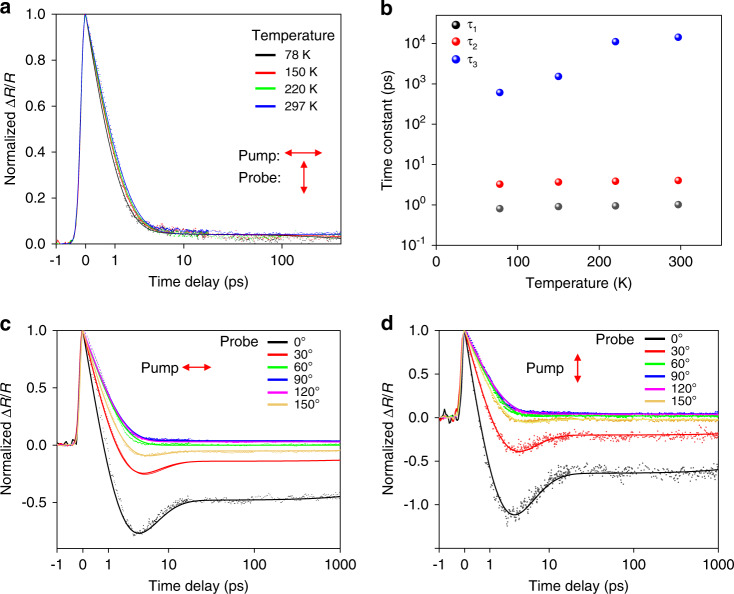
Temperature and probe polarization dependence of transient dynamics. **a** The dependence of Δ*R*/*R* on temperature with cross-polarized pump and probe polarization (0° and 90° for pump and probe, respectively) with 300 μW pump power. **b** The dependence of time constants on temperature. **c**, **d** Probe polarization dependence of normalized Δ*R*/*R* at room temperature with 0° (**c**) and 90° (**d**) pump polarization at room temperature. The pump power is 300 μW. All curves are fitted by tri-exponential function with the same decay constants *τ*_1_ = 1.03 ± 0.01 ps, *τ*_2_ = 3.95 ± 0.03 ps, and *τ*_3_ = 14.0 ± 1.4 ns

Further temperature-dependent studies shown in Fig. 2a, b and pump power-dependent studies (Supplementary S2) reveal the details of the physics processes of the electron–phonon coupling (*τ*_1/2_), as well as the heat dissipation to the substrate(*τ*_3_): all three decay time constants increase as temperature increases, which indicates the strengths of both the electron-phonon coupling (relating to *τ*_1_ and *τ*_2_) and the phonon-phonon coupling to the substrate (relating to *τ*_3_) decrease as temperature increases. On the other hand, the heat dissipation process to the substrate can get slower due to the decrease of the temperature gradient between the lattice and the substrate as the measurement temperature increases. This is because the heat capacity of the lattice increases as the temperature increases, so the elevation of the lattice temperature gets smaller as the measurement temperature increases. Figure 2c, d show the normalized probe polarization-dependent dynamical evolution process (normalized with the peak value at time zero) with pump polarizations along 0° (Fig. 2c) and 90° (Fig. 2d), respectively. The peak values stay positive regardless of the probe polarization, which indicates that the reflection increases along both crystallographic *a* and *b* axes immediately after the photoexcitation. With probe polarization close to crystallographic *b*-axis (60°, 90°, 120°), Δ*R*/*R* stays positive during the whole relaxation process, while with probe polarizations closer to *a*-axis (0°, 30°, 150°), Δ*R*/*R* switches to be negative after a few picoseconds. As shown in the pump polarization-dependent measurements presented later, the anisotropic absorption of light with pump polarization along different crystallographic axes would slightly affect the reflection change when the pump polarization is varied. Although the relaxation curves show very different shapes with probe polarizations around 0° and 90°, we find all relaxation curves can be fit by tri-exponential functions with the common decay time constants: *τ*_1_(1.03 ± 0.01 ps), *τ*_2_(3.95 ± 0.03 ps), and *τ*_3_(14.0 ± 1.4 ns). In Supplementary S3, we show the fitting results with different decay time constants for each probe polarization. We notice the decay time constants are very close to each other and the fitting curves show very limited improvement over those fitted with common decay time constants. This indicates that the relaxation dynamics is isotropic in TaIrTe_4_, independent of the probe polarization. However, due to the anisotropic response of the material, the probe reflection signal strongly depends on the probe polarization. Even the dynamical evolution of hot carrier distribution and lattice temperature stays the same for all directions, the ratio between *A*_*1/2*_ can strongly depend on the probe response difference for different probe polarizations.

**Fig. 3:**
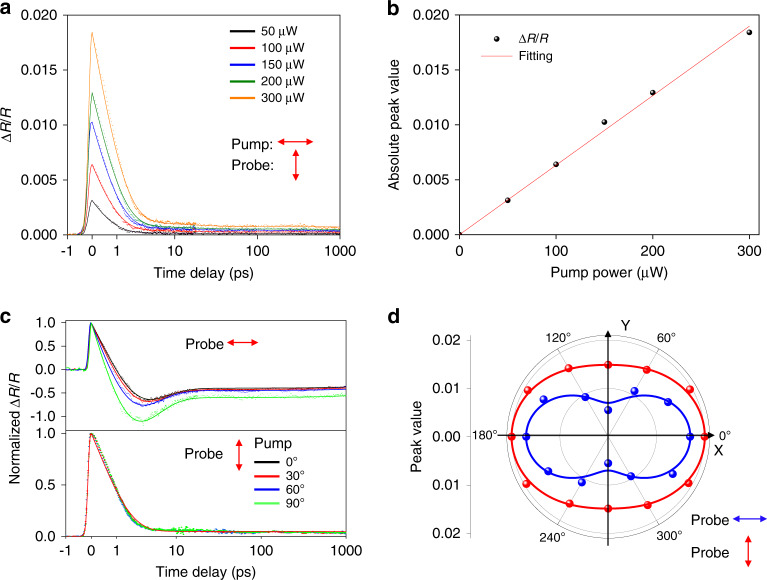
Pump power and polarization dependence of transient dynamics. **a** The dependence of Δ*R*/*R* on pump power with cross-polarized pump and probe polarization (0° and 90° for pump and probe, respectively) at room temperature. **b** The dependence of Δ*R*/*R*|_*t*=0_ on pump power. The experimental data are fit linearly with a red line. **c** Pump polarization dependence of normalized Δ*R*/*R* at room temperature. The pump power is 300 μW. The curves are fitted by Δ*R*/*R* = $A_1e^{ - t/\tau _1} + A_2e^{ - t/\tau _2} + A_3e^{ - t/\tau _3}$ and normalized by the positive peak values at time zero. **d** The dependence of Δ*R*/*R*|_*t*=0_ on pump polarization with 0°(blue) and 90°(red) probe polarization. The results with probe polarization along 0° have been quadrupled for clarity and the fitting equation is: Δ*R*/*R*|_*t*=0_ = *B*_1_cos^2^(*α*_1_) + *B*_2_sin^2^(*α*_1_)

Figure 3 shows the dynamical evolution processes after excitation with different pump powers and polarizations. As the pump power increases, the absolute value of Δ*R*/*R* increases as shown in Fig. 3a. Further quantitative analysis of the three decay time constants obtained from tri-exponential fittings is presented in Supplementary S2, which reveals that only *τ*_1_ and *τ*_2_ increase with pump power, while *τ*_3_ does not show clear pump power dependence. This is quite consistent with the fact that the measured power range is quite limited, which does not change the lattice temperature significantly within the measurement range due to the large lattice heat capacities [See Supplementary S7 for the deduction of the lattice temperature with different excitation powers]. Thus, the effect of pump power on the phonon-phonon coupling to the substrate is relatively weak. On the other hand, the dependence of *τ*_3_ is not very clear due to the relatively large fitting uncertainties. The peak value of Δ*R*/*R* at time zero (Δ*R*/*R*|_*t*=0_) shows a linear dependence on the photoexcited hot carrier density as shown in Fig. 3b. Figure 3c shows the normalized transient reflection spectra as a function of pump polarization. When the probe polarization is 90°, the normalized spectra of different pump polarizations are almost overlapped with each other, however detailed tri-exponential fitting results indicate the decay time constants still have a very weak dependence on the pump polarization angle, similar dependence also applies to 0° probe polarization (see Supplementary S4). On the other hand, (Δ*R*/*R*|_*t*=0_) shows a clear anisotropic response with different pump polarization. Because (Δ*R*/*R*|_*t*=0_) depends linearly on photoexcited hot carrier density as already verified in Fig. 3b, the anisotropic absorption of the pump light dominates the observed pump polarization dependence of the amplitude variance of transient signals and the anisotropic absorption is attributed to the anisotropic crystallographic structure of TaIrTe_4_ in *ab*-plane. Based on that the angle-resolved absorption coefficient *Abs*(*α*_1_) can be calculated by the following equation (See Supplementary S5 for the deduction):

1 $$\begin{array}{ll}{\mathrm{Abs}}(\alpha _1)\\ \approx \frac{{4\sqrt {\varepsilon _1} \left(Re(\sigma _{xx}){\mathrm{cos}}^2\left( {\alpha _1} \right) + Re(\sigma _{yy}){\mathrm{sin}}^2\left( {\alpha _1} \right)\right)}}{{\varepsilon _0c\left( {\sqrt {\varepsilon _2} + \sqrt {\varepsilon _1} } \right)^2}} \\ \propto {\mathrm{{\Delta}}}R/R|_{t = 0}(\alpha _1) = B_1{\mathrm{cos}}^2\left( {\alpha _1} \right) + B_2{\mathrm{sin}}^2\left( {\alpha _1} \right)\end{array}$$

here *α*_1_ denotes the angle between pump polarization and the *x*-axis as marked in Fig. 1c, and it’s assumed that the TaIrTe_4_ sample is sandwiched between SiO_2_ (*ε*_1_) and air (*ε*_2_), *σ*_*xx*_ and *σ*_*yy*_ are a nonzero diagonal component of optical conductivity tensor, *ε*_0_ is the free-space permittivity and *c* is the speed of light. Figure 3d shows the fitting results, it can be determined that the crystallographic *a*-axis of TaIrTe_4_ is along the *x*-axis (0° in Fig. 1c). According to the fitting results in Fig. 3d, we can also get that *Re*(*σ*_*xx*_): *Re*(*σ*_*yy*_) = *B*_1_/*B*_2_ ≈ 1:0.76 for the 2 µm pump wavelength.

**Fig. 4:**
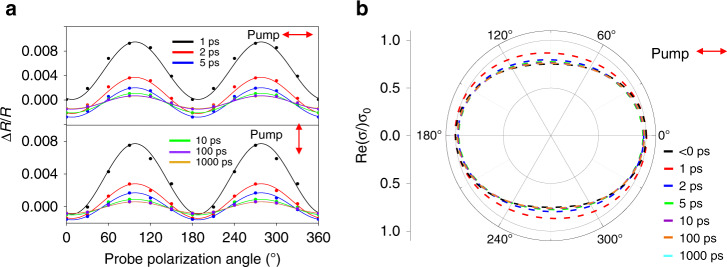
Dynamical evolution of anisotropic response. **a** The dependence of transient reflection signal on the polarization angle of the probe at fixed pump polarization and delay time at room temperature. The pump power is 300 μW. The angular dependence is fitted by Δ*R*/*R* = *C*_1_cos^2^(*α*_2 _− *φ*) + *C*_2_sin^2^(*α*_2_-*φ*), where *α*_2_ is the angle between the probe polarization and *x*-axis as marked in Fig. 1. *φ* is a fitting angle and *φ* *=* 98° from the fitting, Δ*R*/*R* reaches the maximum when *α*_2_ = *φ*. **b** Dynamical evolution of *Re*(*σ*) ellipse, here the pump induced conductivity changes Δ*Re*(*σ*) = *Re*(*σ*)|_pump on_ − *Re*(*σ*)|_pump off_ have been tripled for clarity

Furthermore, if we fix the pump polarization and delay time, and plot the Δ*R*/*R* as a function of probe polarization as shown in Fig. 4a, Δ*R/R* oscillates with probe polarization. The periodic oscillation lasts over the whole relaxation process. Considering that Δ*R/R* is quite small during the whole process, the relation between the optical conductivity change (Δ*σ*) and the experimentally measurable transient reflection signal (Δ*R/R*) can be described by the following equation (see Supplementary S6 for deduction):

2 $$\begin{array}{lll}\qquad\,\,\frac{{{\Delta}R}}{R}\, \approx\, \frac{{4\varepsilon _0c\sqrt {\varepsilon _1} \left[ {\left( {\varepsilon _0c\sqrt {\varepsilon _2} + Re\left( \sigma \right)} \right)^2 - \varepsilon _0^2c^2\varepsilon _1} \right]}}{{\left[ {\left( {\varepsilon _0c\sqrt {\varepsilon _2} + \varepsilon _0c\sqrt {\varepsilon _1} + Re\left( \sigma \right)} \right)^2} \right]\left[ {\left( {\varepsilon _0c\sqrt {\varepsilon _2} - \varepsilon _0c\sqrt {\varepsilon _1} + Re\left( \sigma \right)} \right)^2} \right]}}\\ {\Delta}Re\left( \sigma \right)\,+ \,\frac{{8\varepsilon _0c\sqrt {\varepsilon _1} Im\left( \sigma \right)\left( {\varepsilon _0c\sqrt {\varepsilon _2} + Re\left( \sigma \right)} \right)}}{{\left[ {\left( {\varepsilon _0c\sqrt {\varepsilon _2} + \varepsilon _0c\sqrt {\varepsilon _1} + Re\left( \sigma \right)} \right)^2} \right]\left[ {\left( {\varepsilon _0c\sqrt {\varepsilon _2} - \varepsilon _0c\sqrt {\varepsilon _1} + Re\left( \sigma \right)} \right)^2} \right]}}\\ {\Delta}Im\left( \sigma \right)\,\approx\, \frac{{4\varepsilon _0c\sqrt {\varepsilon _1} \left[\left( {\varepsilon _0c\sqrt {\varepsilon _2} + Re\left( \sigma \right)} \right)^2 - \varepsilon _0^2c^2\varepsilon _1\right]}}{{\left[\left( {\varepsilon _0c\sqrt {\varepsilon _2} + \varepsilon _0c\sqrt {\varepsilon _1} + Re\left( \sigma \right)} \right)^2\right]\left[\left( {\varepsilon _0c\sqrt {\varepsilon _2} - \varepsilon _0c\sqrt {\varepsilon _1} + Re\left( \sigma \right)} \right)^2\right]}}\\ \quad\qquad\qquad ({\Delta}Re(\sigma _{xx}){\mathrm{cos}}^2\alpha _2 + {\Delta}Re(\sigma _{yy}){\mathrm{sin}}^2\alpha _2) \\ \qquad\quad \,\,+\,\frac{{8\varepsilon _0c\sqrt {\varepsilon _1} Im\left( \sigma \right)\left( {\varepsilon _0c\sqrt {\varepsilon _2} + Re\left( \sigma \right)} \right)}}{{\left[ {\left( {\varepsilon _0c\sqrt {\varepsilon _2} + \varepsilon _0c\sqrt {\varepsilon _1} + Re\left( \sigma \right)} \right)^2} \right]\left[ {\left( {\varepsilon _0c\sqrt {\varepsilon _2} - \varepsilon _0c\sqrt {\varepsilon _1} + Re\left( \sigma \right)} \right)^2} \right]}}\\\,\,\,\,\qquad\qquad \left( {({\Delta}Im(\sigma _{xx}){\mathrm{cos}}^2\alpha _2 + {\Delta}Im(\sigma _{yy}){\mathrm{sin}}^2\alpha _2)} \right)\end{array}$$

From Eq. (2), both the real (*Re*(Δ*σ*)) and imaginary (*Im*(Δ*σ*)) part of Δ*σ* contribute to the change of reflection signal. The relationship between Δ*R* and Δ*σ* is a sine function of probe polarization angle (*α*_2_). According to Eq. (2), it requires Δ*σ*_*xx*_ ≠ Δ*σ*_*yy*_ to account for the observed sinusoidal oscillation (Fig. 4a), which implies Δ*σ* must be anisotropic.

Next, we try to extract the conductivity from the experimentally measured transient reflection signal. Although both *Re*(Δ*σ*) and *Im*(Δ*σ*) contribute to Δ*R*, *Re*(Δ*σ*) usually dominates. This can be evaluated according to Eq. (2): the coefficient of the real part (first term of rhs of Eq. 2): $\frac{{4\sqrt {\varepsilon _1} }}{{\varepsilon _0c(\varepsilon _2 - \varepsilon _1)}}\sim \frac{{0.3}}{{Re(\sigma )}}$ is ten times larger than the imaginary part (second term of rhs of Eq. 2): $\frac{{8\sqrt {\varepsilon _1\varepsilon _2} Im\sigma }}{{\varepsilon _0^2c^2(\varepsilon _2 - \varepsilon _1)^2}}\sim \frac{{0.03}}{{Re(\sigma )}}$, taking, $\varepsilon _1 = 1,\varepsilon _2 = 3.9$, $\varepsilon _0c = 3Re(\sigma )$, and $\varepsilon _0c = 9Im(\sigma )$ for 100-nm thick TaIrTe_4_. Considering that Δ*Re*(*σ*) is at least comparable to Δ*Im*(*σ*), the contribution from the imaginary part of Δ*σ* to Δ*R* is negligible, so Δ*Re*(*σ*) can be extracted from Δ*R/R* directly through the following relationship: ${\Delta}R/R \approx \frac{{4\sqrt {\varepsilon _1} {\Delta}{\mathrm{Re}}\sigma }}{{\varepsilon _0c(\varepsilon _2 - \varepsilon _1)}}$, and the dynamic evolution of Δ*Re*(*σ*) obtained from the experimental data is shown in Fig. 4b. We note that the exciting hot carriers lead to an increase in optical conductivity along *y*-axis (Δ*Re*(*σ*_*yy*_) > 0) and a reduction in optical conductivity along *x*-axis (Δ*Re*(*σ*_*xx*_) > 0), which implies a reduction of anisotropic response. The degradation of anisotropy lasts over the whole relaxation process and gradually recovers during the relaxation of photoexcited carriers. In the plot shown in Fig. 4b, we use *Re*(*σ*_*xx*_): *Re*(*σ*_*yy*_) ≈ 1:0.76, which is the experimental value taken from the fitting shown in Fig. 3d for the pump wavelength at 2 μm. The value of complex optical conductivity (*σ*) of bulk TaIrTe_4_ is taken to be (1.7 × 10^3^ – *i* × 5.6 × 10^2^) Ω^−1^/cm according to the infrared spectroscopy experiment^27^. The absolute value *Re*(*σ*_*xx*_):*Re*(*σ*_*yy*_) at 4 μm can be larger according to previous polarization-dependent photocurrent measurement at different excitation wavelength^5^, but this does not affect the qualitative results that the response becomes more isotropic in presence of hot carriers.

The reduction of anisotropic response indicates that the optical response of TaIrTe_4_ becomes more isotropic in the high field transport regime. Here we note the deduced conductivity ellipse may not be valid during the initial stage right after the photoexcitation, this is because the contribution of Δ*Im*(*σ*) cannot be ignored when the photoexcited carriers have not reached the quasi-equilibrium state through rapid electron-electron scattering during this initial stage, so the deduced conductivity ellipse may not closely follow the relation. However, the non-equilibrium state is estimated to be very short because the evolution behavior of the conductivity ellipse becomes qualitatively uniform within a 1 ps delay. After the initial non-equilibrium state and highly excited quasi-equilibrium state, the quasi-equilibrium distribution of the photoexcited carriers mimics the hot carrier distribution in a high field device and is of practical interest for high field electronic and optoelectronic applications. This dynamical evolution of anisotropic response of TaIrTe_4_ is exactly opposite to that of BP^16^: with the presence of hot carriers, the response of BP becomes more anisotropic, while TaIrTe_4_ becomes more isotropic instead. The exact reason for the opposite change of anisotropic response after photoexcitation is not clear, but the opposite responses of these two materials are not unexpected. These two materials have totally different lattice structures that lead to anisotropic response: although the maximum absorption directions are both along *a*-axis, TaIrTe_4_ have zigzag chains ordered in the [Ta-lr-lr-Ta] fashion in crystallographic *a*-axis, while the zigzag chains of BP are in *b*-axis; the band structures of these two materials are also totally different, BP is a semiconductor with energy gap while TaIrTe_4_ is a semimetal without a gap.

In addition, the reduction of the anisotropic response does not depend on the pump polarization. Because the scattering between the hot carriers is rapid, the non-thermal distribution of photoexcited carriers becomes thermalized after the carrier–carrier scattering processes, which is typically within the ~200 fs experimental time resolution. The nonuniform distribution of carriers in *k* space is possibly erased by the rapid carrier–carrier scattering process. This can also explain the isotropic relaxation along with any polarization directions thereafter: although the scattering between phonons and hot carriers and thus the hot carrier relaxation can be anisotropic, the fast carrier–carrier scattering mediates the carrier distribution in *k* space within the experimental time resolution, and this timely mediation of carrier distribution leads to isotropic relaxation behavior magnified in the transient reflection spectroscopy.

## Discussion

The dynamic evolution of the anisotropic response of TaIrTe_4_ unveiled in this work provides indispensable high field device physics that is crucial for optoelectronic devices, such as optical modulators, photodetector, remote sensors, and high field electronic devices based on TaIrTe_4_. On top of the extraordinary photocurrent response of TaIrTe_4_ for photodetection of low energy photons^5,12^, the dynamical response behavior revealed in this work can further extend its applications to fields where high mobility and anisotropy are both pivotal. For instance, high-speed light polarization sensors and ballistic transistors, which require both polarization sensitivity and high-field running capability at the same time. Since the transient energy relaxation of photoexcited carriers is dominated by the scattering during the first few picoseconds, which is ultrafast and thus promises potential applications in ultrafast optoelectronics, such as saturable absorber, optical modulator, and switch, which is capable of working in the challenging long-wavelength region benefiting from semimetal’s gapless nature^28,29^. As a two-dimensional van der Waals material, TaIrTe_4_ can be easily integrated with other layered materials to form various heterostructures, which enables multi-functional devices^30,31^ taking various advantages of individual 2D building blocks. Similar properties may also apply to other layered Weyl semimetals with C_2v_ lattice structure such as Mo(W)Te_2_, though remained to be verified experimentally. This category of materials provides an ideal option that complements BP and ReS_2_ as a polarization-sensitive material to work in the longer wavelength range beyond the energy gap of BP. The weakening of the anisotropic response of TaIrTe_4_ also provides an ideal choice to compensate for the enhancement of the anisotropic response of BP under the high field to keep a constant anisotropic response through material engineering for future high-performance polarization-sensitive optoelectronics and high field electronics.

## Materials and methods

### Sample preparation

A solid-state reaction approach is used to synthesize TaIrTe_4_ single crystals. An argon-filled glove box is used to store the raw materials and the oxygen and moisture levels are well controlled to be less than 0.01 ppm in the glovebox to carry out all manipulations. A flame-sealed quartz tube is pre-loaded with Ta powder (99.99%), Ir powder (99.999%), and Te lump (99.999%) with an atomic ratio of 1:1:12, and then heated in a furnace. The tube was ramped to 1000 °C and stay for about 100 h, then cooled down to 600 °C at a 0.8 °C/h rate. Lastly, the tube was cooled to room temperature. The TaIrTe_4_ single-crystal product is shiny, needle-shaped. A thin flake of TaIrTe_4_ can be exfoliated from the product and transferred onto a 300 nm SiO_2_/Si substrate.

### Transient reflection spectroscopy

A 250 kHz Ti-Sapphire regenerative amplifier (RegA) is used to generate linearly polarized laser pulses at 808 nm (1.53 eV) wavelength with 100 fs pulse width. The 808 nm beam is used as the pump laser of an infrared optical parametric amplifier (OPA) to generate a 1315 nm signal and a 2020 nm idler. Then the signal and the idler are used to pump a difference frequency generator (DFG) to generate 4 µm and used as a probe. The 2020 nm idler is partly used as a pump before the difference frequency generation. The linear polarization of the pump and probe beams are rotated by their respective half waveplates. A 300 mm translation stage is used to adjust the delay time between the pump and probe. A pellicle is used to combine the pump and probe laser and to collect the reflection signal. The combined lasers are focused by a ×40 reflection objective onto the TaIrTe_4_ flake. The spot size of the probe laser is about 8 μm and the spot size of the pump is slightly larger. The reflection signal is detected by an InSb infrared detector with a 2400 nm long pass window. A lock-in amplifier referenced to a mechanical chopper running at 887 Hz is used to read the transient reflection signal of the probe.

## Supplementary information

Supplementary Information for Dynamical Evolution of Anisotropic Response of Type-II Weyl Semimetal TaIrTe4 under Ultrafast Photoexcitation

## Data Availability

The data that support the plots in this paper and other findings of this study are available from the corresponding authors upon reasonable request.
