# Supplementary material for: Dynamical evolution of anisotropic response of type-II Weyl semimetal TaIrTe4 under ultrafast photoexcitation
Source: Light Sci Appl. 2021 May 14;10:101. doi: 10.1038/s41377-021-00546-1 (PMC8121930; doi:10.1038/s41377-021-00546-1)
Supplement: Supplementary file 1 — Supplementary Information for Dynamical Evolution of Anisotropic Response of Type-II Weyl Semimetal TaIrTe4 under Ultrafast Photoexcitation [file 41377_2021_546_MOESM1_ESM.docx]

**Supplementary Information for**

**Dynamical Evolution of Anisotropic Response of Type-II Weyl Semimetal TaIrTe_4_ under Ultrafast Photoexcitation**

Xiao Zhuo^1^,Jiawei Lai^1^, Peng Yu^2,3^,Ze Yu^4^, Junchao Ma^1^,Wei Lu^1,5^, Miao Liu^4,6^, Zheng Liu^3^, Dong Sun^1,7,*^

^1^International Center for Quantum Materials, School of Physics, Peking University, Beijing 100871, China

^2^School of Materials Science and Engineering, Sun Yat-sen University, Guangzhou 510275, Guangdong, China

^3^Centre for Programmed Materials, School of Materials Science and Engineering, Nanyang Technological University, Singapore, 639798, Singapore.

^4^Institute of Physics, Chinese Academy of Sciences, Beijing 100190, China

^5^State Key Laboratory of Precision Measurement Technology and Instruments, School of Precision Instruments and Opto-electronics Engineering, Tianjin University, NO. 92 Weijin Road, Tianjin 300072, China

^6^Songshan Lake Materials Laboratory, Dongguan, Guangdong 523808, China

^7^Collaborative Innovation Center of Quantum Matter, Beijing 100871, China

^*^Addess correspondence to: [sundong@pku.edu.cn](mailto:sundong@pku.edu.cn);

**Table of Contents:**

S1. Comparison between tri-exponential fit and bi-exponential fit

S2. Fitting of power dependence of transient reflection spectra

S3. Fitting of probe polarization dependence of transient reflection spectra

S4. Fitting of pump polarization dependence of transient reflection spectra

S5. Deduction of absorption coefficient *Abs*.

S6. Deduction of transient reflection$\Delta R/R$.

S7. Estimation of the electron and phonon temperature.

S8. Supplementary Reference

**S1. Comparation between tri-exponential fit and bi-exponential fit**

Figure S1 shows the comparison between a tri-exponential fit and a bi-exponential fit with pump polarization at 0° and probe polarization at 90° respectively. It is clear that a tri-exponential decay function fits much better than a bi-exponential decay function, especially for the 2-5 ps delay region, suggesting there are three decay processes contributing to the relaxation of photoexcited carriers.

**
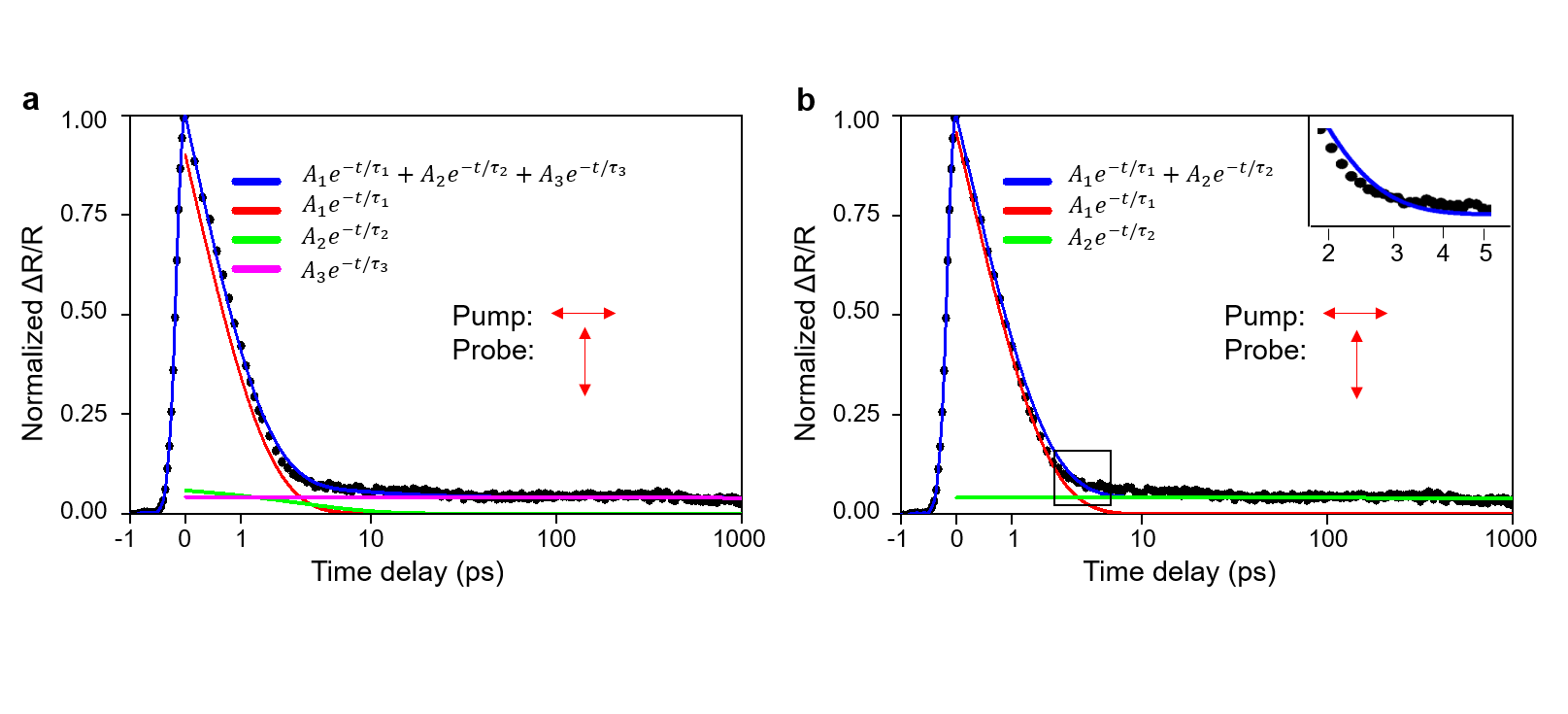
**

**Figure S1**: Comparison between tri-exponential fit and bi-exponential fit with pump polarization at 0° and probe polarization at 90°. The measurement temperature is 297 K and the pump power is 300 μW. (a) Normalized ΔR fitted by tri-exponential function Δ*R/R=*${A_{1}e}^{-t/\tau_{1}}+{A_{2}e}^{-t/\tau_{2}}+{A_{3}e}^{-t/\tau_{3}}$ with *τ*_1_=1.02±0.01 ps, *τ*_2_=4.04±0.05 ps, *τ*_3_=14.0±1.8 ns, *A*_1_=0.90±0.01, *A*_2_=0.058±0.004 and *A*_3_=0.042±0.001. The contributions of each exponential decay components are also plotted separately by lines of different colors. (b) Normalized ΔR fitted by bi-exponential function Δ*R/R=*${A_{1}e}^{-t/\tau_{1}}+{A_{2}e}^{-t/\tau_{2}}$ with *τ*_1_=1.10±0.01 ps, *τ*_2_=14.0±1.9 ns, *A*_1_=0.958±0.01 and *A*_2_=0.042±0.001. The contributions of each exponential decay components are also plotted separately by lines of different colors. Inset shows the zoom-in plot of 2 ps-5ps delay region as marked by black rectangular.

**S2. Pump power dependence of transient reflection spectra**

Figure S2 shows normalized transient reflection spectra with different pump powers. In Figure S2, we use tri-exponential function Δ*R=A*_1_(exp*(-t/τ*_1_) + *A*_2_exp(*-t/τ*_2_) *+ A*_3_exp(-*t/τ*_3_) to fit the data. As the pump power increases, the decay time constant *τ*_1_ and *τ*_2_ increase as shown in Table S1.

| Pump Power(μW) | 50 | 100 | 150 | 200 | 300 |
| --- | --- | --- | --- | --- | --- |
| *τ*_1_(ps) | 0.68±0.01 | 0.78±0.01 | 0.83±0.01 | 0.91±0.01 | 1.02±0.01 |
| *τ*_2_(ps) | 3.11±0.63 | 3.32±0.56 | 3.56±0.52 | 3.75±0.45 | 4.04±0.41 |
| *τ*_3_(ns) | 13.7±3.4 | 14.2±3.1 | 13.6±2.4 | 14.1±2.1 | 14.2±1.8 |
| *A*_1_ | 0.90±0.03 | 0.90±0.02 | 0.90±0.01 | 0.90±0.01 | 0.90±0.01 |
| *A*_2_ | 0.06±0.015 | 0.06±0.011 | 0.06±0.007 | 0.06±0.006 | 0.06±0.004 |
| *A*_3_ | 0.04±0.006 | 0.04±0.004 | 0.04±0.002 | 0.04±0.002 | 0.04±0.001 |

**Table S1**: Decay time constant τ_1_, τ_2_, τ_3_ with different pump excitation powers.

**
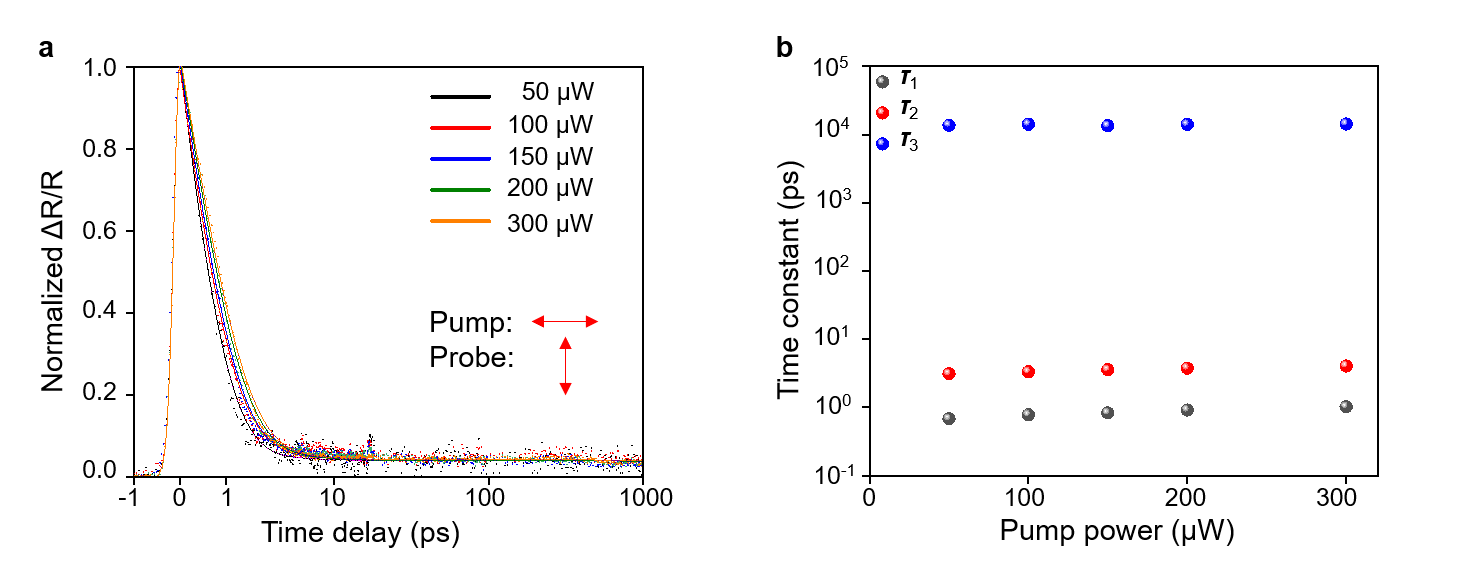
Figure S2**: Pump power dependence of transient reflection spectra. (a) Normalized ΔR with different pump excitation powers (pump fixed at 0° while probe fixed at 90°) at 297K. The solid lines are tri-exponential fittings. (b)Decay time constant *τ*_1_（black), *τ*_2_ (red), *τ*_3_(blue) with different pump excitation powers.

**S3. Fitting of probe polarization dependence of transient reflection spectra**

Figure S3 shows the probe polarization dependence of transient reflection Δ*R* with pump polarization fixed at 0° (Fig. S3a) and 90° (Fig. S3b). The normalized Δ*R* with different probe polarizations are fit with different parameters for individual probe polarizations. The decay parameters are tabulated in Table S2.

| Probe  angle | 0° | 30° | 60° | 90° | 120° | 150° | Parameters in Fig. 2c |
| --- | --- | --- | --- | --- | --- | --- | --- |
| *τ*_1_(ps) | 1.10±0.01 | 1.08±0.01 | 1.02±0.01 | 1.02±0.01 | 1.02±0.01 | 1.05±0.01 | 1.03±0.01 |
| *τ*_2_(ps) | 4.04±0.05 | 4.08±0.06 | 4.03±0.31 | 4.06±0.41 | 4.10±0.34 | 4.06±0.07 | 3.95±0.03 |
| *τ*_3_(ns) | 13.8±2.4 | 13.7±2.3 | 14.2±2.2 | 14.1±1.8 | 14.1±2.5 | 13.7±2.0 | 14.0±1.4 |
| *A*_1_ | 2.21±0.02 | 1.55±0.02 | 1.02±0.01 | 0.90±0.01 | 0.96±0.01 | 1.23±0.02 |  |
| *A*_2_ | -0.76±0.04 | -0.41±0.03 | -0.03±0.004 | 0.06±0.004 | 0.01±0.004 | -0.18±0.01 |  |
| *A*_3_ | -0.45±0.03 | -0.14±0.002 | 0.01±0.001 | 0.04±0.001 | 0.03±0.001 | -0.05±0.002 |  |

**Table S2a**: Fitting parameters for different probe polarizations with pump polarization fixed along 0°. The temperature is 297 K and the pump power is 300 μW.

| Probe  angle | 0° | 30° | 60° | 90° | 120° | 150° | Parameters in Fig. 2d |
| --- | --- | --- | --- | --- | --- | --- | --- |
| *τ*_1_(ps) | 1.01±0.04 | 1.02±0.02 | 0.98±0.01 | 0.96±0.02 | 0.99±0.01 | 1.01±0.02 | 1.03±0.01 |
| *τ*_2_(ps) | 3.77±0.06 | 3.77±0.07 | 3.81±0.35 | 3.92±0.27 | 3.83±0.36 | 3.86±0.07 | 3.95±0.03 |
| *τ*_3_(ns) | 13.8±4.7 | 13.9±3.8 | 14.1±2.4 | 13.9±2.4 | 13.7±2.6 | 14.0±2.8 | 14.0±1.4 |
| *A*_1_ | 3.02±0.08 | 1.72±0.04 | 1.02±0.02 | 0.90±0.02 | 0.95±0.02 | 1.15±0.03 |  |
| *A*_2_ | -1.40±0.12 | -0.50±0.05 | -0.04±0.008 | 0.06±0.009 | 0.03±0.005 | -0.12±0.01 |  |
| *A*_3_ | -0.62±0.09 | -0.22±0.02 | 0.02±0.002 | 0.04±0.002 | 0.04±0.002 | -0.03±0.003 |  |

**Table S2b**: Fitting parameters for different probe polarizations with pump polarization fixed along 90°. The temperature is 297 K and the pump power is 300 μW.

*
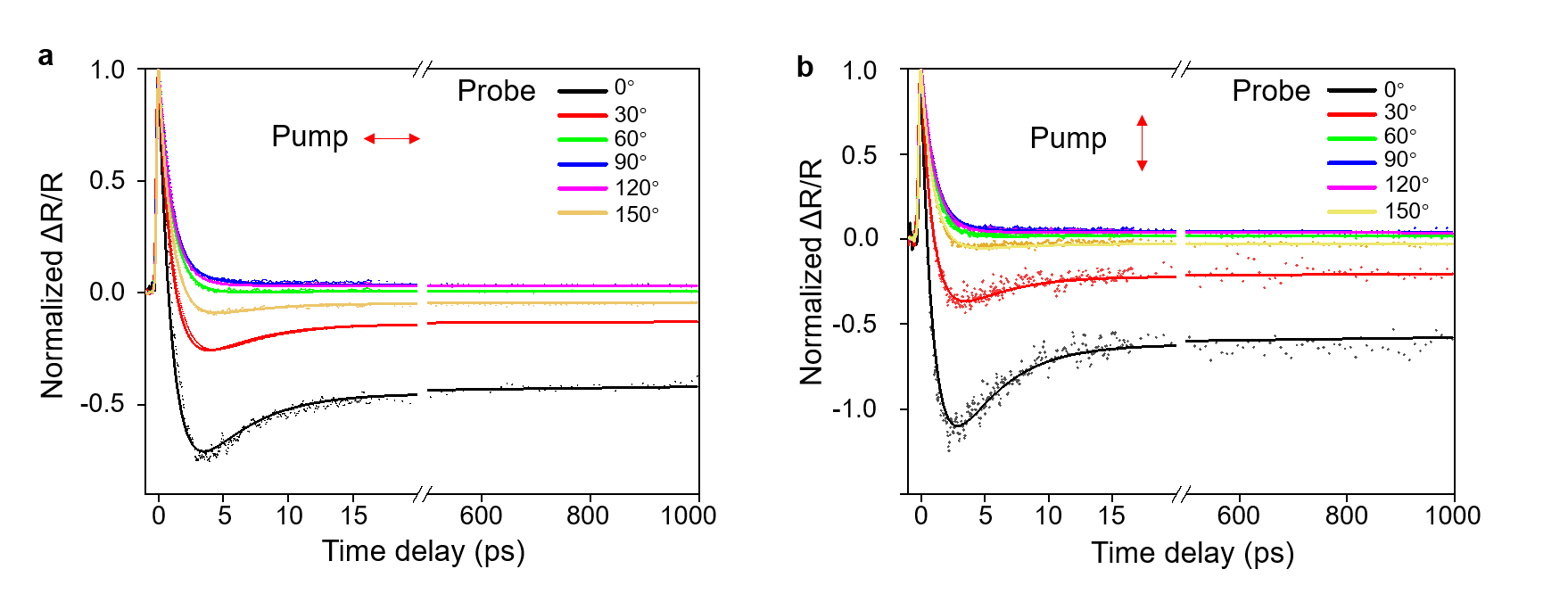
*

**Figure S3**: Tri-exponential fittings of probe polarization dependent transient reflection data taken at room temperature with pump polarization fixed along 0° (a) and 90° (b). The temperature is 297 K and the pump power is 300 μW.

**S4. Fitting of pump polarization dependence of transient reflection spectra**

Figure S4 shows the pump polarization dependence of transient reflection Δ*R* with probe polarization fixed at 0° (Fig. S4a) and 90° (Fig. S4b). Each transient reflection curve is fit with its own best fitting parameters. The decay constants are tabulated in Table S3. It is clear that both *τ*_1_ and *τ*_2_ have weak dependence on pump polarizations and *τ*_3_ is relatively stable, which are similar to the pump power dependence of decay time constants.

| Pump angle | 0° | 30° | 60° | 90° | Parameters in Fig. 3c |
| --- | --- | --- | --- | --- | --- |
| *τ*_1_(ps) | 1.10±0.01 | 1.08±0.01 | 1.05±0.03 | 1.01±0.04 | 1.03±0.01 |
| *τ*_2_(ps) | 4.04±0.05 | 3.99±0.06 | 3.81±0.06 | 3.77±0.06 | 3.95±0.03 |
| *τ*_3_(ns) | 13.8±2.4 | 14.1±2.7 | 14.3±3.9 | 13.8±4.7 | 14.0±1.4 |
| *A*_1_ | 2.21±0.02 | 2.29±0.03 | 2.46±0.06 | 3.02±0.08 |  |
| *A*_2_ | -0.76±0.04 | -0.81±0.05 | -0.95±0.10 | -1.40±0.12 |  |
| *A*_3_ | -0.45±0.03 | -0.48±0.04 | -0.51±0.07 | -0.62±0.09 |  |

**Table S3a**: Fitting parameters for different pump polarizations with probe polarization fixed along 0°. The temperature is 297 K and the pump power is 300 μW.

| Pump angle | 0° | 30° | 60° | 90° | Parameters in Fig. 3d |
| --- | --- | --- | --- | --- | --- |
| *τ*_1_(ps) | 1.02±0.01 | 1.01±0.01 | 0.99±0.01 | 0.96±0.02 | 1.03±0.01 |
| *τ*_2_(ps) | 4.06±0.41 | 4.03±0.28 | 3.99±0.31 | 3.92±0.27 | 3.95±0.03 |
| *τ*_3_(ns) | 14.1±1.8 | 14.1±2.1 | 14.3±2.3 | 13.9±2.4 | 14.0±1.4 |
| *A*_1_ | 0.90±0.01 | 0.90±0.01 | 0.90±0.01 | 0.90±0.02 |  |
| *A*_2_ | 0.06±0.004 | 0.06±0.004 | 0.06±0.005 | 0.06±0.009 |  |
| *A*_3_ | 0.04±0.001 | 0.04±0.001 | 0.04±0.001 | 0.04±0.002 |  |

**Table S3b**: Fitting parameters for different pump polarizations with probe polarization fixed along 90°. The temperature is 297K and the pump power is 300 μW.

**
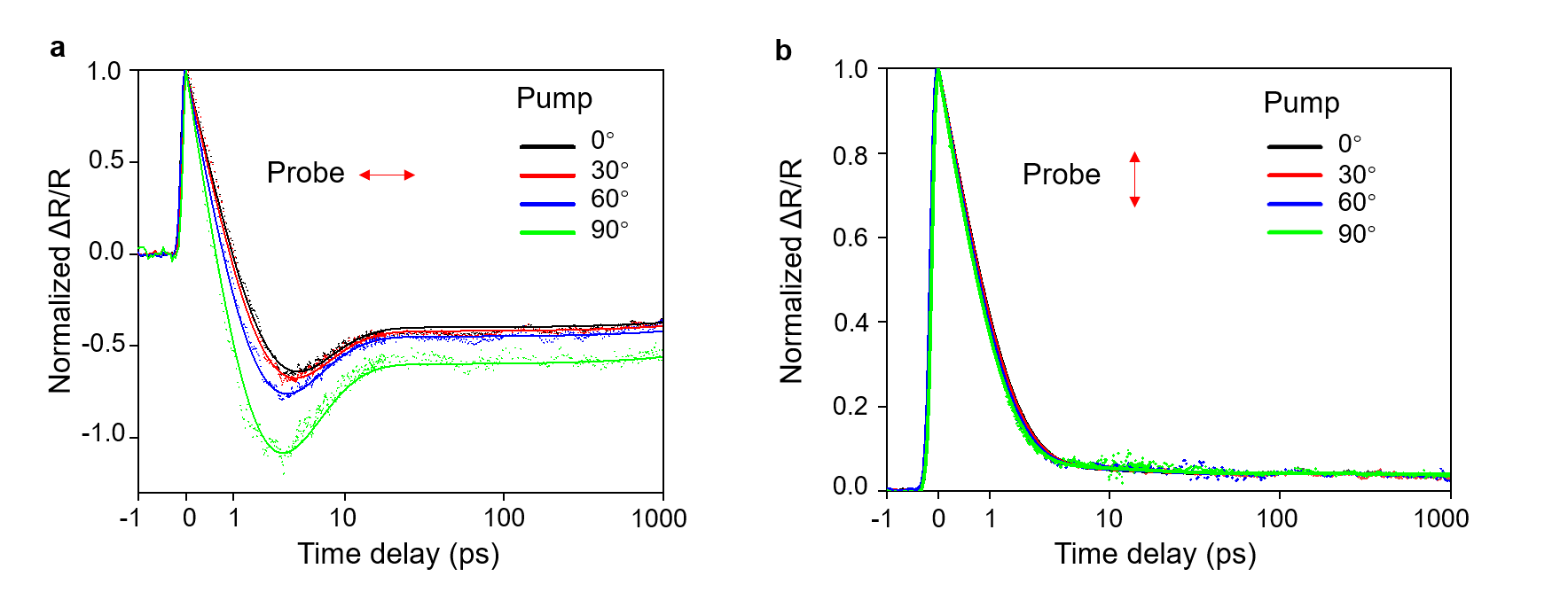
Figure S4**: Triple-exponential fittings of pump polarization dependent transient reflection data taken at room temperature with probe polarization fixed along 0° (a) and 90° (b). The temperature is 297K and the pump power is 300 μW.

**S5. Deduction of absorption coefficient *Abs*.**The reflectivity of the TaIrTe_4_ crystal is given in the literature^1^:

$r=-\frac{\epsilon_{0}c\left( \sqrt{\epsilon_{2}}-\sqrt{\epsilon_{1}} \right)+\sigma_{xx}{cos}^{2}\left( \alpha\right)+\sigma_{yy}{sin}^{2}\left( \alpha\right)}{\epsilon_{0}c\left( \sqrt{\epsilon_{2}}+\sqrt{\epsilon_{1}} \right)+\sigma_{xx}{cos}^{2}\left( \alpha\right)+\sigma_{yy}{sin}^{2}\left( \alpha\right)}$ (1)
where$\epsilon_{0}$ is the free-space permittivity, $\epsilon_{1}$and $\epsilon_{2}$ are the relative permittivity of the air and SiO_2_ in the sample geometry, c is the speed of light, and α is the light polarization angle respect to the *x-* axis of the TaIrTe_4_ crystal, $\sigma$is the optical conductivity of the sample.

The reflection and transmission coefficient *R* and *T* are given by:
*R* = |*r*|^2^ (2)
*T = |1 + r|^2^*$\sqrt{\frac{\epsilon_{2}}{\epsilon_{1}}}$ (3)

So we can get the absorption coefficient *Abs*:
*Abs* = 1 - *T* - *R*

= $\frac{4\epsilon_{0}c\sqrt{\epsilon_{1}}(Re{(\sigma}_{xx}){cos}^{2}\left( \alpha\right)+{Re(\sigma}_{yy}){sin}^{2}\left( \alpha\right))}{{[\epsilon}_{0}c\left( \sqrt{\epsilon_{2}}+\sqrt{\epsilon_{1}} \right)+{Re(\sigma}_{xx}{)cos}^{2}\left( \alpha\right)+Re{(\sigma}_{yy}{)sin}^{2}\left( \alpha\right)]+[{Im(\sigma}_{xx}{)cos}^{2}\left( \alpha\right)+Im{(\sigma}_{yy}{)sin}^{2}\left( \alpha\right)]}$ (4)

Where$\epsilon_{1}=1, \epsilon_{2}=3.9,$ *Re*($\sigma)$ and *Im*($\sigma)$ are real and imaginary part of $\sigma$respectively. According to the infrared spectroscopy experiment^2^, the real part of optical conductivity of bulk TaIrTe_4_ at 4 µm is 1.7*10^3^ Ω^-1^ cm-^1^. The skin depth of TaIrTe_4_ can be obtained from:

*d*=$\sqrt{\frac{2}{\sigma\Omega\mu}}$ (5)

The skin depth of TaIrTe_4_ at 4 μm is 250 nm, which is larger than the sample thickness. From Beer’s law:

*I(z)=I_0_e^-βz^* (6)

*Abs*=$\frac{I_{0}-I(z)}{I_{0}}$ (7)

where *β* is the absorption coefficient, *I*_0_ is the optical intensity at *z=0*, *I*(*z*) is the optical intensity at *z*. From Eq. (6) and (7), the absolute absorption (*Abs*) of 100-nm TaIrTe_4_ sample is about 33%.

The real part of optical conductivity for the TaIrTe_4_ sample can be obtained from^3^

*Re(σ*)=*ϵ*_0_*c***Abs* (8)

for 100-nm TaIrTe_4_ sample,$\epsilon_{0}c\approx3.0Re\left( \sigma\right),$ $\epsilon_{0}c\approx9.0Im\left( \sigma\right),$*Re(σ) <<*${[\epsilon}_{0}c\left( \sqrt{\epsilon_{2}}+\sqrt{\epsilon_{1}} \right)]$*^2^* and (*Im*(*σ*))^2^<< [${[\epsilon}_{0}c\left( \sqrt{\epsilon_{2}}+\sqrt{\epsilon_{1}} \right)$], so the absolute absorption (*Abs*) can be simplified to the following:

$Abs\approx\frac{4\sqrt{\epsilon_{1}}(Re{(\sigma}_{xx}){cos}^{2}\left( \alpha\right)+{Re(\sigma}_{yy}){sin}^{2}\left( \alpha\right))}{\epsilon_{0}c\left( \sqrt{\epsilon_{2}}+\sqrt{\epsilon_{1}} \right)^{2}}$ (9)

**S6. Deduction of transient reflection**

Since *R* = |*r*|^2^, assuming Δ*σ*<<*σ*, which is generally valid for a pump-probe experiment at delay time away from time zero, we can get

$\frac{\Delta R}{R}$*=*$\frac{{-4\epsilon}_{0}c\sqrt{\epsilon_{1}}\Delta Re\left( \sigma\right)[{{Im}^{2}\left( \sigma\right)-\left( \epsilon_{0}c\sqrt{\epsilon_{2}}+Re\left( \sigma\right) \right)}^{2}+{\epsilon_{0}}^{2}c^{2}\epsilon_{1}]}{[{{Im}^{2}\left( \sigma\right)+\left( \epsilon_{0}c\sqrt{\epsilon_{2}}+\epsilon_{0}c\sqrt{\epsilon_{1}}+Re\left( \sigma\right) \right)}^{2}][{{Im}^{2}\left( \sigma\right)+\left( \epsilon_{0}c\sqrt{\epsilon_{2}}-\epsilon_{0}c\sqrt{\epsilon_{1}}+Re\left( \sigma\right) \right)}^{2}]}$

*+*$\frac{{8\epsilon}_{0}c\sqrt{\epsilon_{1}}\Delta Im\left( \sigma\right)Im\left( \sigma\right)\left( \epsilon_{0}c\sqrt{\epsilon_{2}}+Re\left( \sigma\right) \right)}{[{{Im}^{2}\left( \sigma\right)+\left( \epsilon_{0}c\sqrt{\epsilon_{2}}+\epsilon_{0}c\sqrt{\epsilon_{1}}+Re\left( \sigma\right) \right)}^{2}][{{Im}^{2}\left( \sigma\right)+\left( \epsilon_{0}c\sqrt{\epsilon_{2}}-\epsilon_{0}c\sqrt{\epsilon_{1}}+Re\left( \sigma\right) \right)}^{2}]}$ (10)

where $\sigma=\sigma_{xx}\cos^{2}\left( \alpha\right)+\sigma_{yy}\sin^{2}\left( \alpha\right).$ Considering that Δ*σ*<<*σ*, the denominator is a constant, using (*Im(σ*))^2^<< ($\epsilon_{0}c$)^2^,

$\frac{\Delta R}{R}$*=*$\frac{{4\epsilon}_{0}c\sqrt{\epsilon_{1}}\Delta Re\left( \sigma\right)[\left( \epsilon_{0}c\sqrt{\epsilon_{2}}+Re\left( \sigma\right) \right)^{2}-{\epsilon_{0}}^{2}c^{2}\epsilon_{1}]}{[\left( \epsilon_{0}c\sqrt{\epsilon_{2}}+\epsilon_{0}c\sqrt{\epsilon_{1}}+Re\left( \sigma\right) \right)^{2}][\left( \epsilon_{0}c\sqrt{\epsilon_{2}}-\epsilon_{0}c\sqrt{\epsilon_{1}}+Re\left( \sigma\right) \right)^{2}]}$

*+*$\frac{{8\epsilon}_{0}c\sqrt{\epsilon_{1}}\Delta Im\left( \sigma\right)Im\left( \sigma\right)\left( \epsilon_{0}c\sqrt{\epsilon_{2}}+Re\left( \sigma\right) \right)}{[\left( \epsilon_{0}c\sqrt{\epsilon_{2}}+\epsilon_{0}c\sqrt{\epsilon_{1}}+Re\left( \sigma\right) \right)^{2}][\left( \epsilon_{0}c\sqrt{\epsilon_{2}}-\epsilon_{0}c\sqrt{\epsilon_{1}}+Re\left( \sigma\right) \right)^{2}]}$ (11)

If we plug in the values:

$\frac{\Delta R}{R}\approx\frac{0.3}{Re\left( \sigma\right)}\Delta Re\sigma+\frac{0.03}{Re\left( \sigma\right)}\Delta Im\sigma$ (12)

The first term usually dominates when ∆*Re*(*σ*)~ or > ∆*Im*(*σ*).

**S7. Estimation of the electron and phonon temperature.**

We use the two-temperature model to estimate the electron and phonon temperatures. In the two-temperature model, we assume that the electrons absorb energy from the laser and reach the quasi-equilibrium temperature $T_{e}(0)$ after rapid electron-electron within the time resolution of the pump probe measurement. Then, the hot carriers relax through electron-phonon scattering until the lattice and the carriers reach the same equilibrium temperature $T_{L}.$ Under this two-temperature model, the initial electron temperature right after pump excitation（$T_{e}(0)$）and equilibrium lattice temperature（$T_{L}$）can be calculated using the following two equations:

$\int_{300}^{T_{e}(0)} n\gamma TdT$=*Q*Abs* (13)

$\int_{300}^{T_{L}} n(C_{L}+\gamma T)dT$=*Q*Abs* (14)

In the equation (1) and (2), *n≈*5*10^-14^ mol is the amount of TaIrTe_4_ in the excited area taking thickness of 100 nm and excitation spot size of 8 µm, *Q=*1.2 nJ is the energy of single excitation pulse and *Abs=*0.33 is the absorption coefficient calculated from the optical conductivity given in the literature^2^. *γ* ≈ 3 mJ mol^−1^ K^−2^ is the electron heat capacity coefficient of TaIrTe_4_ and $C_{L}$*≈*150 J mol^−1^ K^−1^ is the lattice heat capacity at room temperature. The detailed deduction of the thermodynamic properties is discussed below. From eq. (1) and eq. (2), we can get that the electron temperature after the fast electron-electron scattering is about 2300 K and the lattice temperature after electron-phonon scattering is about 350 K for measurements performed at 300 K. Lattice temperatures with different pump excitation powers are shown in Table.S4:

| Pump power(μW) | 50 | 100 | 150 | 200 | 300 |
| --- | --- | --- | --- | --- | --- |
| Lattice temperature(K) | 308 | 316 | 325 | 334 | 350 |

**Table S4:** Lattice temperatures with different pump excitation powers

The thermodynamic properties of solids can be calculated from DFT. The calculation method is adopted from literatures^4,5^, and the calculation (the free electron model) tells us that the electron heat capacity coefficient *γ* of TaIrTe_4_ is about 3 mJ mol^−1^ K^−2^. The lattice heat capacity can be derived from the Debye model and the Debye temperature from the calculation is about 113.4K. The lattice heat capacity is less sensitive to temperature change in the room temperature region comparing to the low temperature region. And the lattice heat capacity from calculation is $C_{L}$*≈*150 J mol^−1^ K^−1^. The result is close to that of the NbIrTe_4_, which is basically the same structure, but substitute Ta with Nb element. The electron heat capacity coefficient of NbIrTe_4_ is *γ_(NbIrTe4)_*= 2.68 mJ mol^−1^ K^−2^ and the lattice heat capacity $C_{L(NbIrTe4)}$*≈*150 J mol^−1^ K^−1^ according to the experimental observation^6^.

The detailed procedures to calculate the thermodynamic properties (electron heat capacity coefficient *γ*, and the lattice heat capacity $C_{L}$) are shown as followings:

(i). Electron heat capacity coefficient *γ:*

We use the free-electron model to calculate electron heat capacity coefficient. N is the Avogadro constant, $\mathcal{E}_{F}=$7.38 eV is the Fermi energy of TaIrTe_4_, k_B_ is the Boltzmann constant and Z is the number of atoms in TaIrTe_4_.

*γ*=$\frac{NZ\pi^{2}}{2}$ $\frac{k_{B}}{\mathcal{E}_{F}}k_{B}$ (15)

(ii). Lattice heat capacity $C_{L}$:

The lattice heat capacity, according to [Phys. Rev. Materials 3, 073801], is a function of the elasticity of a solid compound. The shear modulus and bulk modulus can be obtained from DFT calculations of TaIrTe_4_, (<https://materialsproject.org/materials/mp-17287/> or DOI:10.17188/1192433), and the Debye temperature of TaIrTe_4_ can be derived from these shear and bulk modulus. The result is shown in Fig. S5. The result is also consistent with the Dulong-Petit law. We have added the estimated electron and lattice temperatures in the final paragraph in page 4. The detailed calculations are added in supplementary information.


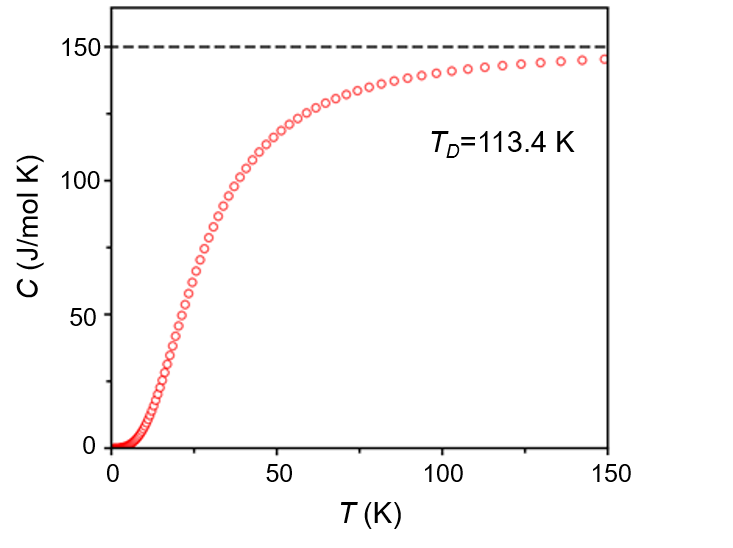


**Figure S5:** Low temperature dependence of lattice heat capacity of TaIrTe_4_.

**S8. References**

1 Low, T. *et al.* Tunable optical properties of multilayer black phosphorus thin films. *Physical Review B* **90**, 075434 (2014).

2 Le Mardelé, F. *et al.* Optical conductivity of the type-II Weyl semimetal TaIrTe_4_. *Physical Review B* **102**, 045201 (2020).

3 Mak, K. F. *et al.* Measurement of the Optical Conductivity of Graphene. *Physical review letters* **101**, 196405 (2008).

4 Nath, P. *et al.* AFLOW-QHA3P: Robust and automated method to compute thermodynamic properties of solids. *Physical Review Materials* **3**, 073801 (2019).

5 Toher, C. *et al.* Combining the AFLOW GIBBS and elastic libraries to efficiently and robustly screen thermomechanical properties of solids. *Physical Review Materials* **1**, 015401 (2017).

6 Zhou, W. *et al.* Nonsaturating Magnetoresistance and Nontrivial Band Topology of Type-II Weyl Semimetal NbIrTe4. **5**, 1900250 (2019).
